# Awake craniotomy for resection of supratentorial glioblastoma: a systematic review and meta-analysis

**DOI:** 10.1093/noajnl/vdaa111

**Published:** 2020-09-18

**Authors:** John J Y Zhang, Keng Siang Lee, Mathew R Voisin, Shawn L Hervey-Jumper, Mitchel S Berger, Gelareh Zadeh

**Affiliations:** 1 Yong Loo Lin School of Medicine, National University of Singapore, Singapore; 2 Bristol Medical School, Faculty of Health Sciences, University of Bristol, Bristol, UK; 3 Department of Neurosurgery, Toronto Western Hospital, University of Toronto, Toronto, Ontario, Canada; 4 Department of Neurological Surgery, University of California, San Francisco, California, USA

**Keywords:** awake craniotomy, extent of resection, glioblastoma, neurological deficit

## Abstract

**Background:**

The goal of glioblastoma (GBM) surgery is to maximize the extent of resection (EOR) while minimizing postoperative neurological complications. Awake craniotomy (AC) has been demonstrated to achieve this goal for low-grade gliomas in or near eloquent areas. However, the efficacy of AC for GBM resection has not been established. Therefore, we aimed to investigate the outcomes of AC for surgical resection of GBM using a systematic review and meta-analysis of published studies.

**Methods:**

Systematic searches of Ovid MEDLINE, Embase, Cochrane Controlled Register of Controlled Trials, and PubMed were performed from database inception to September 14, 2019 for published studies reporting outcomes of AC for GBM resection. Outcome measures analyzed included EOR and the event rate of postoperative neurological deficits.

**Results:**

A total of 1928 unique studies were identified. Fourteen studies reporting 278 patients were included in our meta-analysis. Mean age of patients was 46.9 years (95% confidence interval [CI]: 43.9–49.9). Early and late postoperative neurological deficits occurred in 34.5% (95% CI: 21.9–48.2) and 1.9% (95% CI: 0.0–9.2) of patients, respectively. Pooled percentage of gross total resection (GTR) was 74.7% (95% CI: 66.7–82.1), while the pooled percentage reduction in tumor volume was 95.3% (95% CI: 92.2–98.4).

**Conclusions:**

Limited current evidence suggests that the use of AC for resection of supratentorial GBM is associated with a low rate of persistent neurological deficits while achieving an acceptable rate of GTR. Our findings demonstrate the potential viability of AC in GBM resection and highlight the need for further research on this topic.

**Key Points**

- Awake craniotomy for glioblastoma resection achieves an acceptable GTR rate of 74.7%.
- Neurological deficits persisting beyond 3 months occurred in only 1.9% of patients.

**Importance of the Study**

The goal of glioblastoma (GBM) surgery is to maximize the extent of resection while minimizing postoperative neurological complications. Awake craniotomy (AC) has been demonstrated to achieve this goal for low-grade gliomas in or near eloquent areas. However, the efficacy of AC for GBM resection has not been established. We present the first systematic review and meta-analysis investigating outcomes of AC for GBM specifically. The findings of our study show that the use of AC for resection of supratentorial GBM is associated with a low rate of persistent postoperative neurological deficits while achieving an acceptable rate of GTR. Our findings demonstrate the viability of AC for GBM resection in or near eloquent areas.

Glioblastoma (GBM) is the most common and lethal type of malignant brain tumor. It accounts for 48.3% of all malignant central nervous system tumors in the United States with an annual incidence of 3.22 per 100 000.^1^ Classified by the World Health Organization (WHO) as a grade IV glioma, it is extremely aggressive and possesses the ability to rapidly invade surrounding brain parenchyma. Due to this infiltrative nature, complete surgical resection is rendered near impossible. Coupled with a poor sensitivity to chemo- and radiotherapy, GBM inevitably recurs and is fatal despite the best treatment efforts.^2–4^ The median length of survival following treatment for GBM is approximately 15 months, with only 6.8% of patients surviving beyond 5 years from the time of diagnosis.^1,5–7^

The efficacy of current treatment modalities is limited. Standard of care includes maximal safe surgical resection, chemotherapy, and radiation therapy. Among these, the extent of surgical resection is the most important prognostic factor for GBM patients’ survival.^2,8–10^ Extent of resection (EOR) is largely dependent on the surgeon’s capability to distinguish between normal brain tissue and tumor-invaded parenchyma to maximize resection while preserving neurological function. Awake craniotomy (AC) is an increasingly popular technique used to facilitate this distinction.^11^ During AC, the patient is awake and responsive throughout the duration of tumor excision. This, together with intraoperative cortical and subcortical mapping, aids the surgeon in preventing injury to eloquent areas of the brain.^12,13^ AC has been shown to be superior to craniotomy under general anesthesia (GA) in minimizing the risk of postoperative neurological complications and maximizing EOR for supratentorial brain lesions in or near eloquent areas.^13–15^ However, outcomes of AC for resection of high-grade gliomas including GBM have not been established in the current literature. Only a few studies have reported outcomes associated with the use of AC in GBM patients, with none of these being randomized controlled trials. While there have been 2 previous systematic reviews addressing intraoperative brain mapping for glioma surgery, these studies included both asleep and awake patients as well as low-grade gliomas in their analyses.^16,17^ No systematic reviews have been completed to date on AC in GBM patients. Therefore, we aimed to investigate the outcomes of AC for surgical resection of GBM using a systematic review and meta-analysis of published studies.

## Materials and Methods

This review was conducted in accordance with the Preferred Reporting Items for Systematic Reviews and Meta-Analyses (PRISMA) guidelines.^18^ The study protocol was registered on the PROSPERO International Prospective Register of Systematic Reviews (registration number CRD42019147758).

### Search Strategy

A search string was developed to identify original research studies of AC for supratentorial GBM. The search string comprised synonyms of glioblastoma, glioma, astrocytoma, grade IV, awake craniotomy, intraoperative stimulation, and mapping (Supplementary Table S1). The search was applied to the following 4 electronic databases: Ovid MEDLINE, Embase, Cochrane Central Register of Controlled Trials (CENTRAL), and PubMed. Searches were performed in each database from its inception until September 14, 2019.

### Study Selection

All titles and abstracts were screened independently by 2 reviewers (J.J.Y.Z. and K.S.L.) against a set of predefined eligibility criteria (Supplementary Table S2). Potentially eligible studies were selected for full-text analysis. In the event of multiple publications analyzing the same cohort, the most recent paper was used for evaluation. For studies with outcome data on the use of AC but not specific to GBM, the corresponding authors were contacted for the acquisition of raw data. A response was anticipated for 2 weeks before a decision was made on the eligibility of the study. At each stage, J.J.Y.Z. and K.S.L. reviewed 100% of the screened studies for inclusion to ensure the reliability of study selection. Disagreements were resolved by consensus or appeal to a third senior reviewer (M.R.V.). Agreement between the reviewers on study inclusion was evaluated using Cohen’s kappa.^19^

All original English-language studies reporting EOR or neurological outcome of adult patients undergoing AC for resection of supratentorial GBM were included in our meta-analysis. Studies of small sample sizes were included following recommendations by the Cochrane Statistical Methods Group to not exclude studies purely on the basis of sample size.^20^ Nonetheless, case reports were excluded to reduce the likelihood of publication bias. A minimum sample size of 4 was implemented in accordance with the methodologies of previously published meta-analyses.^21,22^ The quality of included studies was assessed using the Joanna Briggs Institute (JBI) checklist for prevalence studies and the JBI checklist for case series.

### Data Extraction and Outcome Measures

A pro forma was developed to extract data on the following variables: study details, sample size of study, age of included patients, preoperative neurological deficits, tumor volume, eloquent areas, use of preoperative diagnostics such as functional MRI (fMRI), diffusion tensor imaging (DTI), magnetic source imaging (MSI), and navigated transcranial magnetic stimulation (nTMS), and use of intraoperative monitoring techniques such as motor and somatosensory evoked potentials (MEP and SSEP), intraoperative MRI (iMRI), and intraoperative ultrasonography (IOUS).

Primary outcome measures analyzed were EOR and the event rate of postoperative neurological deficits. Secondary outcome measures adopted were 30-day mortality, progression-free survival (PFS), and overall survival (OS).

EOR was reported as either a nonvolumetric, dichotomized outcome or a volumetric percentage, depending on the method of reporting in the included studies. For studies with nonvolumetric EOR reported, the patients were dichotomized into having either gross total resection (GTR) or subtotal resection (STR). GTR was defined according to the authors’ definition. For studies with volumetric EOR reported, the percentage of resection was computed to provide an overall pooled estimate.

Postoperative neurological deficits were categorized in accordance with the classification used in the meta-analysis by De Witt Hamer et al.^16^ Deficits were grouped based on severity (major or minor) and permanency (early or late). Major deficits comprised muscle strength grade 1–3 on the Medical Research Council Scale, aphasia or severe dysphasia, hemianopia, and a vegetative state. All other neurological deficits were considered minor. Minor deficits included but were not limited to grade 4 monoparesis, isolated central facial palsy or other cranial nerve deficit, dysnomia, somatosensory syndrome, and parietal syndrome. Early and late deficits were defined as resolving within 3 months and lasting beyond 3 months after surgery, respectively.

### Statistical Analysis

Meta-analyses of primary endpoints were done assuming the random effects model, which accounts for variance across studies.^23^ Study variance refers to clinical and methodological diversity across studies that arises due to differences in patient characteristics, indications for treatment, treatment methods, surgical techniques, and outcome assessments. Pooled proportions were computed with the inverse variance method using the variance-stabilizing Freeman-Tukey double arcsine transformation.^24^ Confidence intervals (CIs) for individual studies were calculated using the Wilson Score confidence interval method with continuity correction. The *I*^2^ statistic was used to present between-study heterogeneity, where *I*^2^ ≤30%, between 30% and 50%, between 50% and 75%, and ≥75% were considered to indicate low, moderate, substantial, and considerable heterogeneity, respectively.^25^ The *I*^2^ value quantifies the proportion of between-study variation that is attributable to genuine differences in results rather than chance.^26^  *P* values for the *I*^2^ statistic were derived from the chi-squared distribution of Cochran Q test. To identify influential studies or outliers, sensitivity analyses were performed by omitting one study at a time. For pooling of means of numerical variables, we imputed missing means and standard deviations (SDs) from medians and interquartile ranges (IQRs) using the method proposed by Wan et al.^27^

Publication bias was assessed using funnel plots, where an asymmetrical distribution of studies was suggestive of bias.^28^ Quantitative analysis of funnel plot asymmetry was done using Egger’s regression test, based on a weighted linear regression of the treatment effect (expressed as a Freeman-Tukey double arcsine transformed proportion) on its standard error.^29^ Where publication bias was evident, Duval and Tweedie’s trim-and-fill method was adopted to estimate the number of studies missing due to publication bias, augment the observed data, and recompute the summary estimate based on the complete data.^30^

All statistical analyses were performed using R software version 3.4.3 (R Foundation for Statistical Computing, 2016). *P* values less than .05 were considered statistically significant.

## Results

### Baseline Study Characteristics

**Figure 1.**
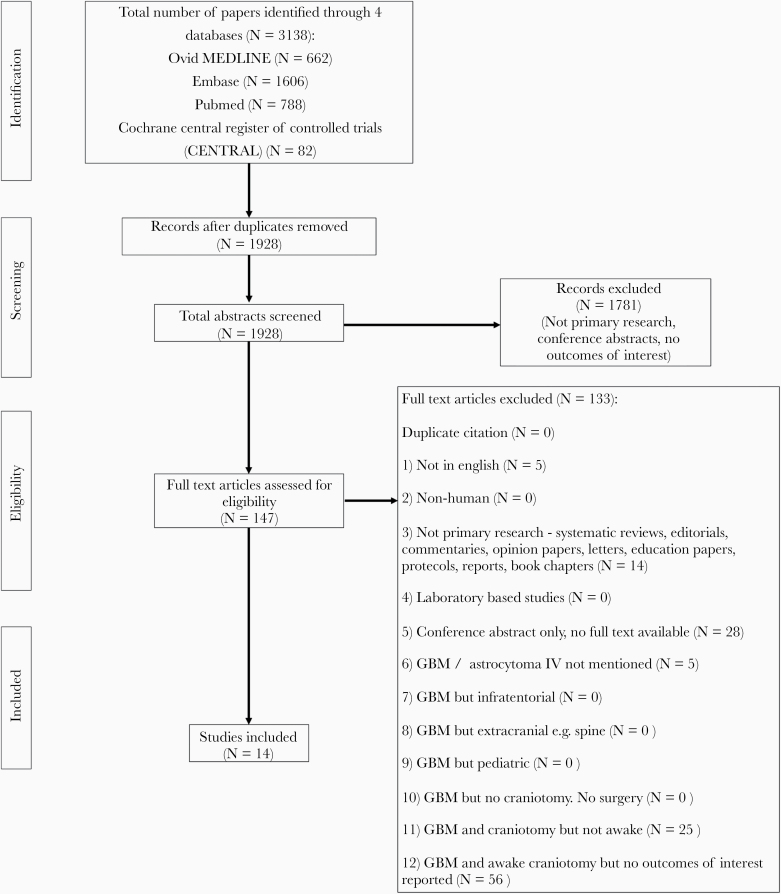
PRISMA flow diagram for study selection.

Our search yielded 1928 unique publications after removal of duplicates. After screening of titles and abstracts, 147 publications were reviewed in full text. A total of 14 studies reporting 278 patients were eventually included for our meta-analysis (Figure 1).^31–44^ Reliability of study selection between observers was substantial at both the title and abstract screening stage (Cohen’s *κ* = 0.75) and the full-text review stage (Cohen’s *κ* = 0.71).^19^ All included studies were retrospective. Using the JBI checklist for prevalence studies, 12 studies attained a full score of 9 and 2 studies attained a score of 8 (Supplementary Table S3). Using the JBI checklist for case series, 11 studies attained a full score of 10, 1 study attained a score of 9, and 2 studies attained a score of 8 (Supplementary Table S4).

**Figure 2.**
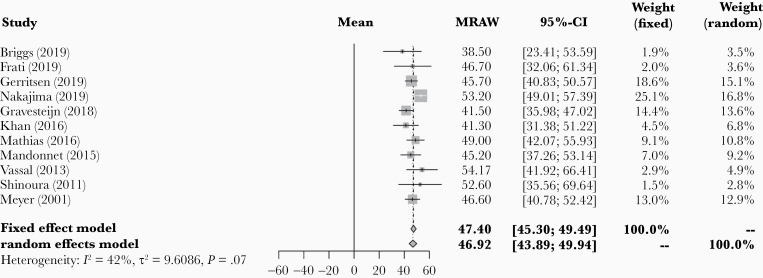
Forest plot of pooled mean age of included patients.

Mean and SD of age were reported in 11 of the 14 included studies across 134 patients. Pooled mean age across these 11 studies was 46.9 years (95% CI: 43.9–49.9; Figure 2). Study heterogeneity was not statistically significant (*I*^2^ = 41.6% [95% CI: 0.0–71.2], *P* = .072). Preoperative tumor volume was reported in 8 studies across 89 patients. Pooled mean tumor volume was 35.1 cm^3^ (95% CI: 21.7–48.5; Supplementary Figure S1). Study heterogeneity for tumor volume was considerable (*I*^2^ = 84.0% [95% CI: 70.2–91.4], *P* < .001). Preoperative neurological deficits were reported in 3 studies and were present in all 16 patients in these 3 studies. Proximity of the resected tumor to eloquent areas was reported in 13 of the 14 included studies. In these 13 studies, 264 out of 272 patients (97.1%) had a tumor located in or near an eloquent area. Table 1 summarizes the baseline characteristics and patient outcomes in each included study.

**Table 1.** Summary of Included Studies

Author, Year	Sample Size	Age (years)	Preoperative Tumor Volume (cm^3^)	Use of Subcortical Mapping	Volumetric Extent of Resection (%)	Gross Total Resection	Early Neurological Deficit	Late Neurological Deficit
Briggs et al., 2019^31^	4	38.5 ± 15.4	46.0 ± 32.8	Yes	74.6 ± 17.1	NR	1 (25%)	0
Frati et al., 2019^32^	6	46.7 ± 18.3	26.3 ± 15.4	Yes	NR	5 (83.3%)	1 (16.7%)	0
Gerritsen et al., 2019^33^	37	45.7 ± 15.1	66.3 ± 64.3	Yes	94.9 ± 10.6	NR	16 (43.2%)	3 (8.1%)
Nakajima et al., 2019^34^	30	53.2 ± 11.7	NR	Yes	97.0 ± 8.7	NR	NR	NR
Pichierri et al., 2019^35^	6	NR	47.0 ± 38.0	Yes	NR	4 (66.7%)	NR	NR
Gravesteijn et al., 2018^36^	5	41.5 ± 6.3	NR	No	71.8 ± 24^a^	NR	NR	NR
Khan et al., 2016^37^	6	41.3 ± 12.4	NR	No	NR	NR	NR^b^	NR^b^
Mathias et al., 2016^38^	9	49.0 ± 10.6	NR	Yes	NR	7 (77.8%)	3 (33.3%)	0
Mandonnet et al., 2015^39^	13	45.2 ± 14.6	27.2 ± 32.5	Yes	99.7 ± 0.8	NR	NR	NR
Vassal et al., 2013^40^	6	54.2 ± 15.3	34.5 ± 12.7	Yes	97.0 ± 6.1	NR	1 (16.7%)	0
Shinoura et al., 2011^41^	5	52.6 ± 19.4	NR	No	NR	2 (40%)	NR	NR
Kim et al., 2009^42^	134	NR	NR	No	NR	98 (73.1%)	NR	NR
Low et al., 2007^43^	4	NR	41.8 ± 26.1	Yes	87.5 ± 8.1	NR	NR	NR
Meyer et al., 2001^44^	13	46.6 ± 10.7	10.3 ± 11.7	No	97.5 ± 6.6	NR	NR	NR

NR, not reported. All numerical data reported as mean ± standard deviation. All categorical outcome data reported as *n* (%).

^a^Imputed from median and interquartile range.

^b^One case of postoperative mild right facial weakness which persisted till discharge was reported, but the permanency of deficit was unknown.

The use of preoperative diagnostics was reported in 4 studies. fMRI was used in 18 patients and DTI was used in 16 patients. There was no reported use of MSI or nTMS. The use of intraoperative monitoring adjuncts was reported in 9 studies. MEP or SSEP was used in 179 patients and iMRI was used in 28 patients. There was no reported use of IOUS.

### Neurological Outcome

**Figure 3.**
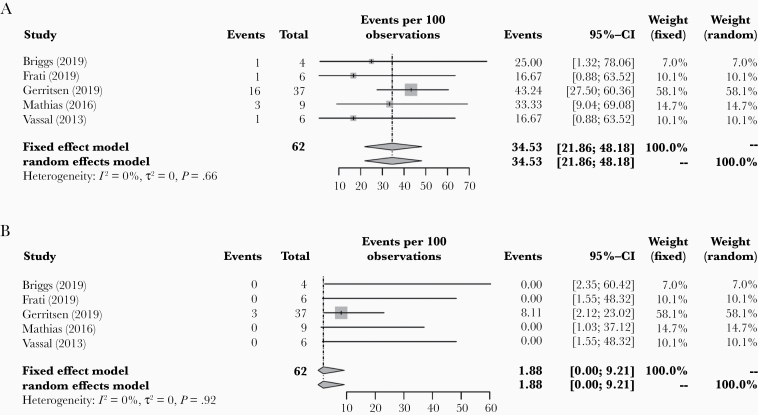
Forest plots of pooled (A) early and (B) late postoperative neurological deficits in included patients.

Neurological outcome was reported in 6 of the included studies. Timing of neurological deficits (early or late) was specified in 5 of the 6 studies, in a total of 62 patients. Pooled early neurological deficit rate was 34.5% (95% CI: 21.9–48.2; Figure 3). Study heterogeneity was negligible (*I*^2^ = 0.0% [95% CI: 0.0–65.5], *P* = .660). When each of the 5 studies was omitted one at a time, pooled early neurological deficit rate ranged from 23.5% to 36.7%.

Publication bias for early neurological deficits was statistically significant using Egger’s regression test (*P* = .035; Supplementary Figure S2). Using the trim-and-fill method, 3 additional studies were computed, giving an adjusted pooled early neurological deficit rate of 40.1% (95% CI: 28.5–52.8). Adjusted study heterogeneity was negligible (*I*^2^ = 0.0% [95% CI: 0.0–63.0], *P* = .525). Severity of early neurological deficits was specified in 18 patients. Twelve patients had minor deficits and 6 patients had major deficits.

Pooled late neurological deficit rate was 1.9% (95% CI: 0.0–9.2) in the 62 patients with the timing of deficit reported (Figure 3). Study heterogeneity was negligible (*I*^2^ = 0.0% [95% CI: 0.0–12.8], *P* = .917). When each of the 5 studies was omitted one at a time, pooled late neurological deficit rate ranged from 0.0% to 2.6%. There was no evidence of publication bias for late neurological deficits demonstrated using Egger’s regression test (*P* = .069; Supplementary Figure S2). Severity of late neurological deficits was specified in 3 patients. One patient had a minor deficit and 2 patients had major deficits.

### Extent of Resection

**Figure 4.**
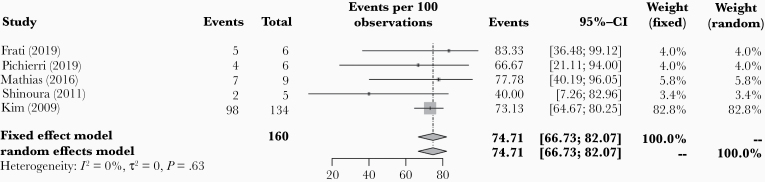
Forest plot of pooled percentage of gross total resection in included patients.

EOR was reported in a total of 13 studies. In 5 of the 13 studies, the number of patients with GTR was specified. In a total of 160 patients, pooled percentage of GTR was 74.7% (95% CI: 66.7–82.1; Figure 4). Study heterogeneity was negligible (*I*^2^ = 0.0% [95%CI: 0.0–68.1], *P* = .625). When each of the 5 studies was omitted one at a time, pooled GTR rate ranged from 70.1% to 75.5%. There was no evidence of publication bias for GTR demonstrated using Egger’s regression test (*P* = .453; Supplementary Figure S3).

**Figure 5.**
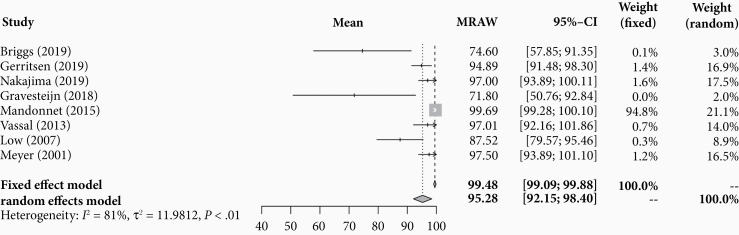
Forest plot of pooled volumetric extent of resection in included patients.

Eight studies reported volumetric EOR. Across 112 patients, pooled mean percentage reduction in tumor volume was 95.3% (95% CI: 92.2–98.4; Figure 5). Study heterogeneity was considerable (*I*^2^ = 80.8% [95% CI: 63.0–90.0], *P* < .001).

### Survival Outcome

There were no deaths within 30 days after surgery in the 6 studies that reported 30-day mortality. PFS and OS were reported in 5 studies, but the reported data were insufficient or too heterogenous to compute pooled mean estimates. Pichierri et al.^35^ reported an average PFS of 21 months in 6 GBM patients. Among these 6 patients, all 4 patients who underwent GTR had OS of more than 25 months, while the 2 patients who underwent STR died at 12 and 30 months. In the study by Briggs et al.,^31^ 2 out of 4 GBM patients were disease-free at 11 months after surgery, while the other 2 had died as a result of tumor progression. Gerritsen et al.^33^ reported a median OS of 17 months in a sample of 37 patients. Of the 5 GBM patients reported by Gravesteijn et al.,^36^ 2 had died at 12-month follow-up and 3 had died at 24 months after surgery. Meyer et al.^44^ reported a mean OS of 13.3 months in a sample of 13 patients.

### AC Versus Craniotomy Under GA

Four studies analyzed and compared an AC arm with a “Craniotomy under GA” arm. EOR was significantly higher with AC than craniotomy under GA in the study by Gerritsen et al.^33^ (mean EOR 94.89% for AC vs 70.30% for craniotomy under GA, *P* = .0001), but did not achieve statistical significance in the other 3 studies. The study by Gerritsen et al. was also the only one to find a lower rate of late minor postoperative complications with AC than craniotomy under GA. Survival outcome was similar between AC and craniotomy under GA in the studies by Gerritsen et al.^33^ and Gravesteijn et al.,^36^ but higher for the AC group in Pichierri et al.^35^ (4 out of 6 patients alive at an average of 25 months’ follow-up for the AC group vs 1 out of 8 patients alive at 26 months in the GA group). Notably, both groups in the study by Pichierri et al. also had iMRI used. Long-term functional status was evaluated only in the study by Nakajima et al.,^34^ which showed significantly higher Karnofsky Performance Scale (KPS) scores in the AC group compared to the GA group. Of these studies, the study by Gerritsen et al.^33^ was the only one that controlled for tumors in eloquent areas.

## Discussion

Our systematic review and meta-analysis demonstrated a pooled early neurological deficit rate of 34.5% (40.1% after adjusting for possible publication bias) and late neurological deficit rate of 1.9% across patients who underwent AC for GBM. Pooled GTR rate was 74.7%, with a pooled mean volumetric EOR of 95.9%. To the best of our knowledge, this is the first study to systematically assess the outcomes of AC specifically for GBM patients using a comprehensive meta-analysis.

### Neurological Outcome

A key takeaway from our study is that while there may be a relatively higher rate of early neurological deficits in the first 3 months after surgery, these deficits appear to be temporary, as the rate of deficits that persist beyond 3 months is minimal. All patients who had early postoperative deficits within 3 months of surgery were reassessed after 3 months, with none being lost to follow-up due to disease progression or other reasons. This is in keeping with the meta-analysis of intraoperative stimulation mapping (ISM) for all types of gliomas by De Witt Hamer et al.,^16^ in which there was a substantially higher early neurological deficit rate of 47.9% compared to the late neurological deficit rate of 6.4%. A number of factors likely contributed to this finding. First, AC involves intraoperative mapping through cortical and subcortical electrostimulation. Such stimulation works by reversible modulation of populations of neurons within and around the tumor thereby causing transient sensorimotor or behavioral changes. This process creates functional cortical and subcortical maps within the framework of an individual patient’s anatomy. The resultant transient deficits may persist from a few weeks to 3 months after surgery.^16^ Second, immediate postoperative neurological deficits after AC can largely be attributed to cerebral edema and manipulation and retraction of tissue. A diagnosis of GBM is also significantly associated with a higher risk of intraoperative brain swelling, which may account for intraoperative seizures or worsening of neurologic deficits.^45^ Third, as almost all of our included patients had a tumor located in or near an eloquent area, the presence of resection-induced contusion, edema, and hypoperfusion adjacent to the resection cavity could easily contribute to the significant rate of early neurological deficits. It is unknown whether the deficits presented in our meta-analysis are ischemic in nature as none of the included studies reported postoperative assessment of patients using diffusion-weighted MRI (DWI). Similarly, it was not possible to draw conclusions on the severity of neurological deficits given the small sample of patients with severity data reported. Nonetheless, a substantial proportion of postoperative deficits were temporary and resolved within a short span of 3 months after the surgery.

Several surgical adjuncts could be adopted during AC to optimize postoperative neurological outcome. These include intraoperative testing methods that play a critical role in achieving reliable cortical localization. For example, visual object naming is a specific task designed to assess the presence and degree of anomia, a very common symptom in many aphasic subjects.^46^ Its use is important in accurate identification of the language area given that there can be a high degree of individual variability in language localization.^47^ Subcortical mapping, in addition to cortical mapping, is another valuable intraoperative technique. Compared to cortical localization, identification of subcortical functional pathways may pose a greater challenge due to our relatively poorer understanding of subcortical anatomical and functional connectivity.^48^ Furthermore, there is a lack of established functional neuroimaging techniques for subcortical brain structures. While advances in DTI have allowed for better preoperative visualization of subcortical tracts, DTI provides only anatomical and not functional information and may be limited in areas where the tracts pass through the tumor or edema.^49–52^ For these reasons, ISM remains the gold standard for accurate localization of subcortical pathways. The use of subcortical mapping for both language and motor pathways has been demonstrated to be beneficial in reducing postoperative neurological complications.^48,52–54^ In our meta-analysis, 9 out of 14 included studies reported the use of subcortical mapping. However, we were unable to test for its association with postoperative neurological deficits as all the studies that reported neurological outcomes also reported the use of subcortical mapping.

Besides intraoperative adjuncts, several technical nuances aimed at reducing postoperative neurological deficits have also been described. Studies have purported that the distance between the point of electrostimulation and the vital functional structures of the brain is associated with the permanency of postoperative neurological deficits.^55,56^ Strategies to increase this distance include using a higher electrical current and pressing the stimulator firmly against the wall of the resection cavity to widen the safety margin. To reduce the risk of hypoperfusion and ischemia, subpial dissection while paying careful attention to vascular structures, coupled with postoperative assessment of cerebral perfusion using DWI, may prove beneficial.^57,58^ Other methods to minimize postoperative deficits include perioperative corticosteroids to reduce edema and intensive physical therapy after surgery.

### Extent of Resection

While an optimal neurological outcome is critical to preserve quality of life after surgery, tumor control with maximal safe resection remains the main goal of surgery. In the current molecular era, there is growing interest in the interplay between EOR and the molecular subtype of gliomas. A recent multicenter study by Molinaro et al.^59^ clarified this by demonstrating improved OS associated with maximal resection of contrast-enhanced tumor, regardless of isocitrate dehydrogenase 1 or 2 gene (IDH)-wild or IDH-mutant subtype and methylation status of the promoter region of the DNA repair enzyme *O*^6^-methylguanine-DNA methyltransferase (MGMT).^60^ In fact, in younger patients below the age of 65 years, surgical resection of even the non-contrast-enhancing disease was shown to confer a greater survival benefit. Similarly, in a recent study of 1204 GBM patients, Al-Holou et al.^61^ advocated the use of perilesional rather than intralesional resection to ensure removal of all contrast-enhancing components of the tumor while minimizing neurological deficits. The importance of maximizing tumor resection as outlined in these studies is precisely the reason why AC plays a critical role in GBM surgery. In patients who underwent AC for GBM resection, our meta-analysis demonstrated a GTR rate of 74.7%. This is comparable to the 79.1% GTR rate reported in the meta-analysis of ISM for high-grade gliomas by Gerritsen et al., and the 74.9% GTR rate demonstrated in the meta-analysis of ISM for all types of gliomas by De Witt Hamer et al.^16^ The acceptable pooled GTR rate from our meta-analysis suggests that the low rate of persistent postoperative neurological deficits in patients who underwent AC for GBM resection did not come at the price of reduced EOR.

Despite its benefits, the use of AC is not without its risks, hence the decision to perform AC should be deliberated prudently. The risk of an intraoperative seizure has been reported to be higher in patients undergoing AC for resection of intraaxial tumors and tumors of the supplementary motor area.^62,63^ Intraoperative seizure is a known risk factor for procedure failure, reduced rate of GTR, and higher incidence of short-term postoperative motor and speech deterioration.^62^ Additionally, elderly patients undergoing AC tend to have a longer length of stay than their younger counterparts, which may be related to their reduced tolerability of the procedure.^64^ The patients included in our meta-analysis had a mean age of 46.9 years (95% CI: 43.9–49.9), which may not be representative of older GBM patients (median age in the general cohort of GBM patients is 65 years).^1^ Our meta-analysis demonstrated a low rate of permanent neurological deficits despite a substantial proportion of patients (97.1%) having tumors located in or near eloquent areas. Therefore, considering the above risks of AC, we recommend the use of AC specifically for tumors in or near the eloquent areas of the brain. Tumors located in or near speech areas typically warrant the use of AC, but ISM under GA could be a safe alternative for other eloquent areas such as motor areas.^65^ With that said, there has been growing recognition that the execution of a motor function involves not only muscle contraction, but also a combination of sensory feedback and higher cortical functions that can only be appreciated under awake conditions.^66^

To safely maximize EOR in glioma surgery, several technologies besides AC have been developed in recent years. These include iMRI, IOUS, and fluorescence-guided surgery.^67–69^ In a recent meta-analysis of patients with high-grade gliomas, Eljamel et al.^70^ showed that the above intraoperative modalities attained comparable rates of GTR, with a slightly higher GTR rate for the fluorescein-guided resections. The GTR rate for patients allocated to the fluorescence-guided surgery group, 5-ALA fluorescence-guided surgery group, IOUS, and iMRI groups was 84.4%, 69.1%, 73.4%, and 70%, respectively. However, there is a paucity of well-established evidence demonstrating the EOR outcomes of these intraoperative adjuncts in GBM resection specifically.

### AC Versus Craniotomy Under GA

Several studies have sought to compare the outcomes of AC with craniotomy under GA for tumors located in eloquent areas. In a retrospective case-control study of 58 patients with perirolandic, eloquent, motor area gliomas, Eseonu et al.^71^ identified a higher rate of GTR, shorter length of hospital stay, and higher KPS scores in the AC group than the GA group. A recent meta-analysis corroborated these findings by demonstrating a higher mean EOR with AC than with GA in a total of 2351 glioma patients who had tumors located near or in motor areas of the brain.^72^ However, these studies also included low-grade gliomas hence their findings may not be applicable to GBM specifically. Our systematic review identified 4 studies that directly compared outcomes of AC and craniotomy under GA for GBM patients. Conclusions drawn from these studies must be judiciously interpreted given their small sample sizes and retrospective nature. The study by Gerritsen et al. was the only one that adjusted for eloquent tumors. In this retrospective matched case-control study, a greater EOR and lower rate of late minor postoperative complications were observed in patients who had undergone AC. However, the authors were unable to show a clear survival benefit between the AC and GA groups. The recent large analysis by Molinaro et al.^59^ revealed a median OS of 14.2 months in 761 surgical GBM patients. In comparison, the limited survival data of AC for GBM identified in our systematic review showed survival rates comparable to if not better than that reported by Molinaro et al. Such a comparison, however, is greatly limited and could be confounded by many patient and treatment factors that were unaccounted for. Therefore, whether the benefits conferred by AC in safely maximizing EOR translate into higher survival rates down the road for GBM patients remains a question that needs to be addressed.

### Limitations

Limitations of our meta-analysis, beyond the specific ones that have been mentioned with their respective findings, include the retrospective nature of the included studies and the heterogeneity among them. Due to the small number of suitable studies, our ability to perform certain analyses including meta-regression for the exploration of possible confounders was limited. In addition, a few of the included studies had small sample sizes, which may have introduced publication bias and exacerbated the file-drawer problem. To minimize the extent of these limitations, we performed sensitivity analyses to attempt to identify outlier studies. Finally, our results must be interpreted bearing in mind that the patients included in our analysis were relatively young with a mean age of 46.9 years. This may not be reflective of the majority of GBM patients, who have a median age of 65 years.^1^

### Future Directions

Moving forward, initial steps include small prospective studies designed to determine the safety of AC in patients with GBM. Ultimately, a randomized controlled trial or large prospective cohort study is needed in order to account for the bias and confounders that exist in retrospective studies, one of which is currently underway.^73^ The findings from our meta-analysis contribute to addressing this gap in the literature at present and highlight a need for more rigorous data from prospective studies.

## Conclusions

Current evidence on the use of AC for supratentorial GBM resection is largely limited to small retrospective studies. Within these studies, AC achieves both an acceptable rate of GTR and a low rate of persistent neurological deficits. To our knowledge, this is the first systematic review and meta-analysis examining the role of AC in GBM specifically. The results of this study illustrate the potential feasibility of AC for patients with supratentorial GBM in or near eloquent areas and emphasize the need for future prospective studies to better determine its efficacy and outcomes.

## Supplementary Material

vdaa111_suppl_Supplementary_Material

## Notes

**Authorship statement**. Study conception and design: J.J.Y.Z. and G.Z. Acquisition of data: J.J.Y.Z., K.S.L., and M.R.V. Analysis and interpretation of data: J.J.Y.Z., K.S.L., M.R.V., S.L.H., M.S.B., and G.Z. Manuscript writing: J.J.Y.Z., K.S.L., M.R.V., S.L.H., M.S.B., and G.Z. Reviewed and approved the final version of the manuscript: J.J.Y.Z., K.S.L., M.R.V., S.L.H., M.S.B., and G.Z. Study supervision: G.Z.

There are no unpublished papers cited in this manuscript.
