# Supplementary material for: Awake craniotomy for resection of supratentorial glioblastoma: a systematic review and meta-analysis
Source: Neurooncol Adv. 2020 Sep 18;2(1):vdaa111. doi: 10.1093/noajnl/vdaa111 (PMC7542985; doi:10.1093/noajnl/vdaa111)
Supplement: vdaa111_suppl_Supplementary_Material [file vdaa111_suppl_supplementary_material.docx]

Supplementary Table S1. Full search phrases used for the 4 respective databases.

| **Pubmed** | | | 788 articles |
| --- | --- | --- | --- |
| (((((iv[Text Word]) OR (four[Text Word])) AND (grade[Text Word])) AND ((“Glioma”[Mesh]) OR (glioma[Text Word]) OR (“Astrocytoma”[Mesh]) OR (astrocytoma[Text Word]))) OR (glioblastoma[Text Word]) OR (“Glioblastoma”[Mesh])) AND ((((scalp[Text Word]) OR (“Scalp”[Mesh])) AND (block*[Text Word])) OR (awake[Text Word]) OR (“Electric Stimulation”[Mesh]) OR (stimulation[Text Word]) OR (“Brain Mapping”[Mesh]) OR (“Intraoperative Neurophysiological Monitoring”[Mesh]) OR (“Monitoring, Intraoperative”[Mesh]) OR (intra-op*[Text Word]) OR (intraop*[Text Word])) AND ((“Craniotomy”[Mesh]) OR (craniotomy[Text Word]) OR (resect*[Text Word]) OR (surg*[Text Word]) OR (“Neurosurgery”[Mesh]) OR (neurosurg*[Text Word]) OR (“Neurosurgical Procedures”[Mesh])) | | | |
| **Ovid MEDLINE** | | | 662 articles |
| Glioblastoma concept | | | |
| 1 | iv.tw. | | |
| 2 | four.tw. | | |
| 3 | 1 or 2 | | |
| 4 | grade.tw. | | |
| 5 | 3 and 4 | | |
| 6 | exp Glioma/ | | |
| 7 | glioma.tw. | | |
| 8 | exp Astrocytoma/ | | |
| 9 | astrocytoma.tw. | | |
| 10 | 6 or 7 or 8 or 9 | | |
| 11 | 5 and 10 | | |
| 12 | exp Glioblastoma/ | | |
| 13 | glioblastoma.tw. | | |
| 14 | 11 or 12 or 13 | | |
| Awake brain stimulation concept | | | |
| 15 | scalp.tw. | | |
| 16 | exp Scalp/ | | |
| 17 | 15 or 16 | | |
| 18 | block*.tw. | | |
| 19 | 17 and 18 | | |
| 20 | awake.tw. | | |
| 21 | exp Electric Stimulation | | |
| 22 | stimulation.tw. | | |
| 23 | exp Brain Mapping/ | | |
| 24 | exp Intraoperative Neurophysiological Monitoring/ or exp Monitoring, Intraoperative/ | | |
| 25 | intra-op*.tw. | | |
| 26 | intraop*.tw. | | |
| 27 | 19 or 20 or 21 or 22 or 23 or 24 or 25 or 26 | | |
| Neurosurgery concept | | | |
| 28 | exp Craniotomy/ | | |
| 29 | craniotomy.tw. | | |
| 30 | resect*.tw. | | |
| 31 | surg*.tw. | | |
| 32 | exp Neurosurgery/ | | |
| 33 | neurosurg*.tw. | | |
| 34 | exp Neurosurgical Procedures/ | | |
| 35 | 28 or 29 or 30 or 31 or 32 or 33 or 34 | | |
| 36 | 14 and 27 and 35 | | |
| **Embase** | | | 1606 articles |
| **Glioblastoma concept** | | | |
| 1 | iv.tw. | | |
| 2 | four.tw. | | |
| 3 | 1 or 2 | | |
| 4 | grade.tw. | | |
| 5 | 3 and 4 | | |
| 6 | exp glioma/ | | |
| 7 | glioma.tw. | | |
| 8 | exp astrocytoma/ | | |
| 9 | astrocytoma.tw. | | |
| 10 | 6 or 7 or 8 or 9 | | |
| 11 | 5 and 10 | | |
| 12 | exp glioblastoma/ | | |
| 13 | glioblastoma.tw. | | |
| 14 | 11 or 12 or 13 | | |
| **Awake brain mapping concept** | | | |
| 15 | scalp.tw. | | |
| 16 | exp scalp/ | | |
| 17 | 15 or 16 | | |
| 18 | block*.tw. | | |
| 19 | 17 and 18 | | |
| 20 | awake.tw. | | |
| 21 | exp stimulation/ or exp electrical brain stimulation test/ | | |
| 22 | stimulation.tw. | | |
| 23 | exp brain Mapping/ | | |
| 24 | exp intraoperative neurophysiological monitoring/ or exp intraoperative monitoring/ | | |
| 25 | intra-op*.tw. | | |
| 26 | intraop*.tw. | | |
| 27 | 19 or 20 or 21 or 22 or 23 or 24 or 25 or 26 | | |
| **Neurosurgery concept** | | | |
| 28 | exp craniotomy/ | | |
| 29 | craniotomy.tw. | | |
| 30 | resect*.tw. | | |
| 31 | surg*.tw. | | |
| 32 | exp Neurosurgery/ | | |
| 33 | neurosurg*.tw. | | |
| 34 | 28 or 29 or 30 or 31 or 32 or 33 | | |
| 35 | 14 and 27 and 34 | | |
| **Cochrane Controlled Register of Trials CENTRAL** | | 82 articles | |
| **Glioblastoma concept** | | | |
| 1 | (iv):ti,ab,kw | | |
| 2 | (four):ti,ab,kw | | |
| 3 | #1 OR #2 | | |
| 4 | (grade):ti,ab,kw | | |
| 5 | #3 AND #4 | | |
| 6 | MeSH descriptor: [Glioma] explode all trees | | |
| 7 | (glioma):ti,ab,kw | | |
| 8 | MeSH descriptor: [Astrocytoma] explode all trees | | |
| 9 | (astrocytoma):ti,ab,kw | | |
| 10 | #6 OR #7 OR #8 OR #9 | | |
| 11 | #5 and #10 | | |
| 12 | MeSH descriptor: [Glioblastoma] explode all trees | | |
| 13 | (glioblastoma):ti,ab,kw | | |
| 14 | #11 or #12 OR #13 | | |
| **Awake brain mapping concept** | | | |
| 15 | MeSH descriptor: [Scalp] explode all trees | | |
| 16 | (scalp):ti,ab,kw | | |
| 17 | #15 OR #16 | | |
| 18 | (block*):ti,ab,kw | | |
| 19 | #17 AND #18 | | |
| 20 | (awake):ti,ab,kw | | |
| 21 | MeSH descriptor: [Electric Stimulation] explode all trees | | |
| 22 | (stimulation):ti,ab,kw | | |
| 23 | MeSH descriptor: [Brain Mapping] explode all trees | | |
| 24 | MeSH descriptor: [Intraoperative Neurophysiological Monitoring] explode all trees | | |
| 25 | MeSH descriptor: [Monitoring, Intraoperative] explode all trees | | |
| 26 | (intra-op*):ti,ab,kw | | |
| 27 | (intraop*):ti,ab,kw | | |
| 28 | #19 OR #20 OR #21 OR #22 OR #23 OR #24 OR #25 OR #26 OR #27 | | |
| **Neurosurgery concept** | | | |
| 29 | MeSH descriptor: [Craniotomy] explode all trees | | |
| 30 | (craniotomy):ti,ab,kw | | |
| 31 | (resect*):ti,ab,kw | | |
| 32 | (surg*):ti,ab,kw | | |
| 33 | MeSH descriptor: [Neurosurgery] explode all trees | | |
| 34 | (neurosurg*):ti,ab,kw | | |
| 35 | MeSH descriptor: [Neurosurgical Procedures] explode all trees | | |
| 36 | #29 OR #30 OR #31 OR #32 OR #33 OR #34 OR #35 | | |
| 37 | #14 AND #28 AND #36] | | |

Supplementary Table S2: Inclusion and exclusion criteria used to select studies for the review

| Inclusion criteria | Exclusion criteria |
| --- | --- |
| - Any peer-reviewed, published interventional or observational study reporting awake resection of supratentorial GBM - At least 4 human adult participants of ages 18 and above - Clinical diagnosis of GBM or grade 4 glioma or grade 4 astrocytoma - Reports either a neurological outcome or extent of resection, or both | - Not written in English - Not a primary research e.g. narrative and systematic reviews, editorials, commentaries, opinion papers, letters, education papers, conference abstracts, protocols, reports, theses or book chapter. Case series with <4 GBM patients with awake craniotomy. - Non-human subjects (e.g. murine, porcine studies) - Laboratory based, not carried out in clinical setting e.g. tumor or blood samples from humans tested within laboratory - Low-grade or grade 3 gliomas - Infratentorial tumours - Extracranial tumours e.g. spine tumours, - Paediatric patients (age < 18), - Patients who had asleep surgery or surgery under general anesthesia - GBM and awake craniotomy but does not report extent of resection or post-op neurological outcome |

Supplementary Table S3. Joanna Briggs Institute quality assessment checklist for prevalence studies.

|  | **Question no.** | | | | | | | | | |  |
| --- | --- | --- | --- | --- | --- | --- | --- | --- | --- | --- | --- |
| **Study** | **1** | **2** | **3** | **4** | **5** | **6** | **7** | **8** | **9** | **Overall** | |
| Briggs (2019) | ✔ | ✔ | ✔ | ✔ | ✔ | ✔ | ✔ | ✔ | ✔ | ✔ | |
| Frati (2019) | ✔ | ✔ | ✔ | ✔ | ✔ | ✔ | ✔ | ✔ | ✔ | ✔ | |
| Gerritsen (2019) | ✔ | ✔ | ✔ | ✔ | ✔ | ✔ | ✔ | ✔ | ✔ | ✔ | |
| Nakajima (2019) | ✔ | ✔ | ✔ | ✔ | ✔ | ✔ | ✔ | ✔ | ✔ | ✔ | |
| Pichierri (2019) | ✔ | ✔ | ✔ | ✔ | ✔ | ✔ | ✔ | ✔ | ✔ | ✔ | |
| Gravesteijn (2018) | ✔ | ✔ | ✔ | ✔ |  | ✔ | ✔ | ✔ | ✔ | ✔ | |
| Khan (2016) | ✔ | ✔ | ✔ | ✔ |  | ✔ | ✔ | ✔ | ✔ | ✔ | |
| Mathias (2016) | ✔ | ✔ | ✔ | ✔ | ✔ | ✔ | ✔ | ✔ | ✔ | ✔ | |
| Mandonnet (2015) | ✔ | ✔ | ✔ | ✔ | ✔ | ✔ | ✔ | ✔ | ✔ | ✔ | |
| Vassal (2013) | ✔ | ✔ | ✔ | ✔ | ✔ | ✔ | ✔ | ✔ | ✔ | ✔ | |
| Shinoura (2011) | ✔ | ✔ | ✔ | ✔ | ✔ | ✔ | ✔ | ✔ | ✔ | ✔ | |
| Kim (2009) | ✔ | ✔ | ✔ | ✔ | ✔ | ✔ | ✔ | ✔ | ✔ | ✔ | |
| Low (2007) | ✔ | ✔ | ✔ | ✔ | ✔ | ✔ | ✔ | ✔ | ✔ | ✔ | |
| Meyer (2001) | ✔ | ✔ | ✔ | ✔ | ✔ | ✔ | ✔ | ✔ | ✔ | ✔ | |

1. Was the sample frame appropriate to address the target population?

2. Were study participants sampled in an appropriate way?

3. Was the sample size adequate?

4. Were the study subjects and the setting described in detail?

5. Was the data analysis conducted with sufficient coverage of the identified sample?

6. Were valid methods used for the identification of the condition?

7. Was the condition measured in a standard, reliable way for all participants?

8. Was there appropriate statistical analysis?

9. Was the response rate adequate, and if not, was the low response rate managed

appropriately?

Supplementary Table S4. Joanna Briggs Institute quality assessment checklist for case series.

|  |  | | **Question no.** | | | | | | | | |  |
| --- | --- | --- | --- | --- | --- | --- | --- | --- | --- | --- | --- | --- |
| **Study** | **1** | **2** | | **3** | **4** | **5** | **6** | **7** | **8** | **9** | **10** | **Overall** |
| Briggs (2019) | ✔ | ✔ | | ✔ | ✔ | ✔ | ✔ | ✔ | ✔ | ✔ | ✔ | ✔ |
| Frati (2019) | ✔ | ✔ | | ✔ | ✔ | ✔ | ✔ | ✔ | ✔ | ✔ | ✔ | ✔ |
| Gerritsen (2019) | ✔ | ✔ | | ✔ | ✔ | ✔ | ✔ | ✔ | ✔ | ✔ | ✔ | ✔ |
| Nakajima (2019) | ✔ | ✔ | | ✔ | ✔ | ✔ | ✔ | ✔ | ✔ | ✔ | ✔ | ✔ |
| Pichierri (2019) | ✔ | ✔ | | ✔ | ✔ | ✔ | ✔ | ✔ | ✔ | ✔ | ✔ | ✔ |
| Gravesteijn (2018) | ✔ | ✔ | | ✔ | ✔ | ✔ | ✔ | ✔ | ✔ | ✔ | ✔ | ✔ |
| Khan (2016) | ✔ | ✔ | |  | ✔ | ✔ | ✔ | ✔ | ✔ | ✔ | ✔ | ✔ |
| Mathias (2016) | ✔ | ✔ | | ✔ |  |  | ✔ | ✔ | ✔ | ✔ | ✔ | ✔ |
| Mandonnet (2015) | ✔ | ✔ | | ✔ | ✔ | ✔ | ✔ | ✔ | ✔ | ✔ | ✔ | ✔ |
| Vassal (2013) | ✔ | ✔ | | ✔ | ✔ |  | ✔ | ✔ | ✔ | ✔ |  | ✔ |
| Shinoura (2011) | ✔ | ✔ | | ✔ | ✔ | ✔ | ✔ | ✔ | ✔ | ✔ | ✔ | ✔ |
| Kim (2009) | ✔ | ✔ | | ✔ | ✔ | ✔ | ✔ | ✔ | ✔ | ✔ | ✔ | ✔ |
| Low (2007) | ✔ | ✔ | | ✔ | ✔ | ✔ | ✔ | ✔ | ✔ | ✔ | ✔ | ✔ |
| Meyer (2001) | ✔ | ✔ | | ✔ | ✔ | ✔ | ✔ | ✔ | ✔ | ✔ | ✔ | ✔ |

1. Were there clear criteria for inclusion in the case series?

2. Was the condition measured in a standard, reliable way for all participants included in the

case series?

3. Were valid methods used for identification of the condition for all participants included in the

case series?

4. Did the case series have consecutive inclusion of participants?

5. Did the case series have complete inclusion of participants?

6. Was there clear reporting of the demographics of the participants in the study?

7. Was there clear reporting of clinical information of the participants?

8. Were the outcomes or follow up results of cases clearly reported?

9. Was there clear reporting of the presenting site(s)/clinic(s) demographic information?

10. Was statistical analysis appropriate?


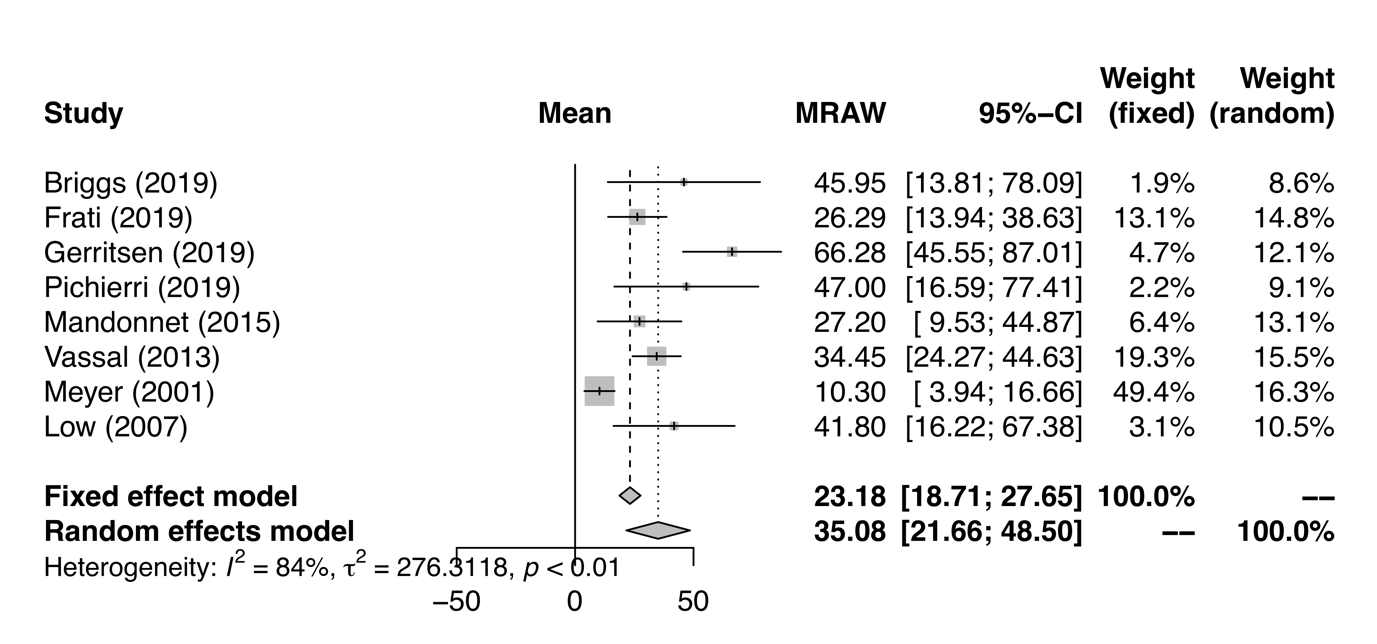


Supplementary Figure S1. Forest plot of pooled mean preoperative tumor volume in included patients.


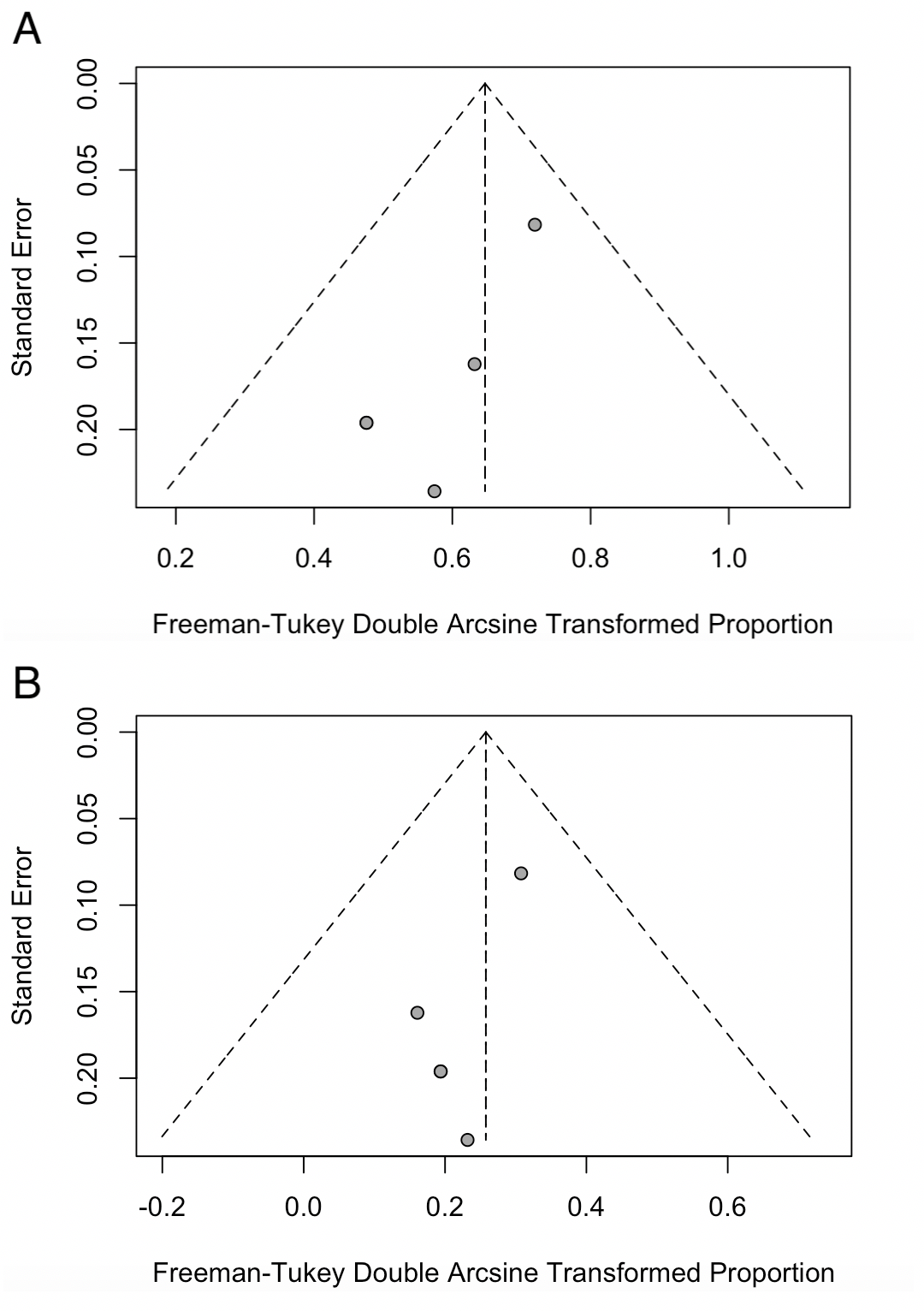


Supplementary Figure S2. Funnel plots for (A) early and (B) late postoperative neurological deficits across studies.


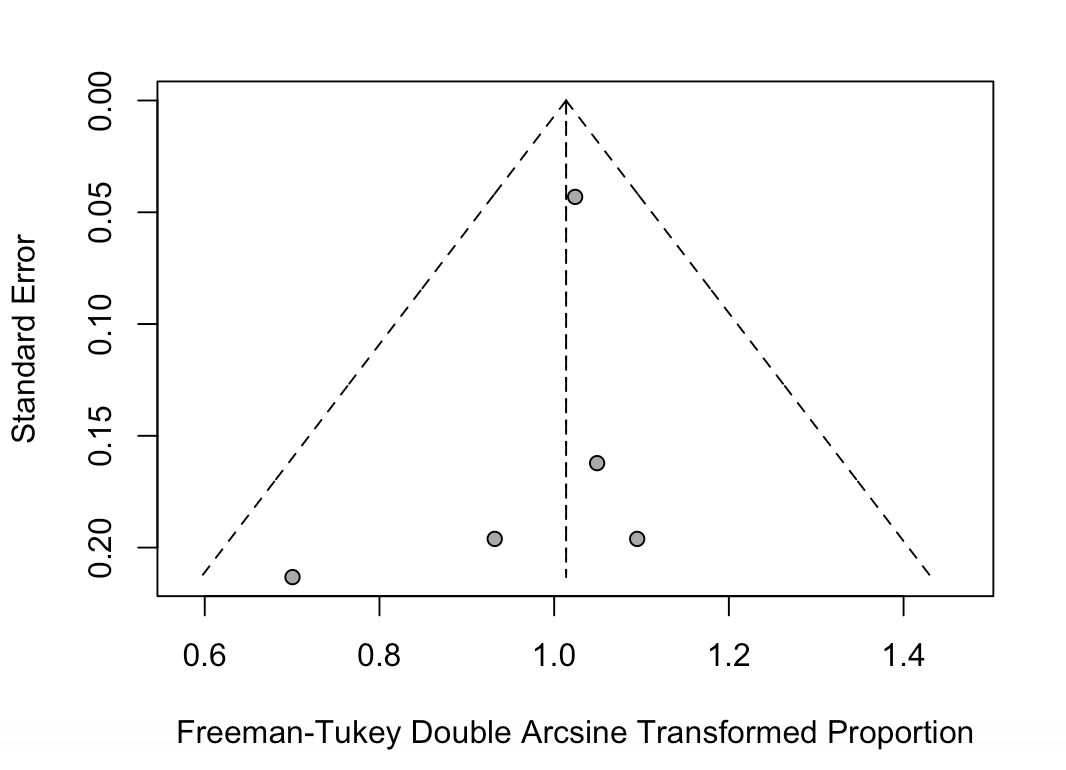


Supplementary Figure S3. Funnel plot for percentage of gross total resection across studies.
